# Cardiac magnetic resonance imaging in systemic sclerosis: a cross-sectional observational study of 52 patients

**DOI:** 10.1136/ard.2008.095836

**Published:** 2008-11-28

**Authors:** A-L Hachulla, D Launay, V Gaxotte, P de Groote, N Lamblin, P Devos, P-Y Hatron, J-P Beregi, E Hachulla

**Affiliations:** 1 Department of Cardiovascular Radiology, Regional University Hospital, Lille 2 University, Lille, France; 2 Department of Internal Medicine, National Reference Centre for Systemic and Autoimmune Rare Diseases (Scleroderma), Regional University Hospital, Lille 2 University, Lille, France; 3 Department of Cardiology, Regional University Hospital, Lille 2 University, Lille, France; 4 Department of Statistics, Regional University Hospital, Lille 2 University, Lille, France

## Abstract

**Objectives:**

To assess the prevalence and patterns of cardiac abnormalities as detected by cardiac magnetic resonance imaging (MRI) in systemic sclerosis (SSc).

**Methods:**

Fifty-two consecutive patients with SSc underwent cardiac MRI to determine morphological, functional, perfusion at rest and delayed enhancement abnormalities.

**Results:**

At least one abnormality on cardiac MRI was observed in 39/52 patients (75%). Increased myocardial signal intensity in T2 was observed in 6 patients (12%), thinning of left ventricle (LV) myocardium in 15 patients (29%) and pericardial effusion in 10 patients (19%). LV and right ventricle (RV) ejection fractions were altered in 12 patients (23%) and 11 patients (21%), respectively. LV diastolic dysfunction was found in 15/43 patients (35%). LV kinetic abnormalities were found in 16/52 patients (31%) and myocardial delayed contrast enhancement was detected in 11/52 patients (21%). No perfusion defects at rest were found. Patients with limited SSc had similar MRI abnormalities to patients with diffuse SSc. Seven of 40 patients (17%) without pulmonary arterial hypertension had RV dilatation.

**Conclusions:**

This study shows that MRI is a reliable and sensitive technique for diagnosing heart involvement in SSc and for analysing its mechanisms, including its inflammatory, microvascular and fibrotic components. Compared with echocardiography, MRI appears to provide additional information by visualising myocardial fibrosis and inflammation. RV dilatation appeared to be non-specific for pulmonary arterial hypertension but could also reflect myocardial involvement related to SSc. Further studies are needed to determine whether cardiac MRI abnormalities have an impact on the prognosis and treatment strategy.

Heart involvement in systemic sclerosis (SSc) affects the prognosis of the disease when it is clinically evident.^1^ Myocardial fibrosis is the pathological hallmark of this complication and has been reported in 50–80% of cases in necropsy studies, whereas it is rarely clinically obvious.^2^ ^3^ The main limitation of the usual methods of assessing heart involvement (echocardiography, perfusion scan) is that they are not specific for myocardial fibrosis.^4^ ^5^ Cardiac magnetic resonance imaging (MRI) is a recent, accurate and sensitive method of studying heart structure and function non-invasively and precisely.^6^ ^7^ Previous studies have shown that MRI is helpful in the diagnosis of acute inflammatory myocarditis^8^ and myocardial fibrosis.^6^ ^9^ Three studies have recently assessed the usefulness of cardiac MRI in SSc, focusing either on delayed contrast enhancement abnormalities,^6^ on ventricular volumes and ejection fractions^10^ or on perfusion index.^11^ The aims of our study were to perform a comprehensive analysis of cardiac MRI in SSc and to compare the cardiac MRI findings according to the clinical features including cutaneous extension and the presence of pulmonary arterial hypertension (PAH).

## Methods

### Patients

Fifty-two consecutive unselected patients followed up at the Reference Centre for Scleroderma in Lille, France and fulfilling the American College of Rheumatology criteria for the diagnosis of SSc^12^ and/or LeRoy’s classification criteria for SSc^13^ were enrolled in this cross-sectional observational study.

Clinical assessment collected data on age at onset of the first symptom of SSc except Raynaud’s phenomenon, age at onset of Raynaud’s phenomenon and cutaneous extension graded according to the LeRoy classification.^13^ Overt coronary arterial disease was excluded based on clinical examination and a systematic ECG.

All patients underwent Doppler echocardiography (Philips Sonos 5500; Philips Medical Systems, Andover, Massachusetts, USA) by a senior cardiologist (PDG) within 1 month before or after MRI. PAH was suspected in patients with a peak velocity of tricuspid regurgitation (VTR) >2.5–3 m/s and unexplained dyspnoea, or with VTR >3 m/s and warranted confirmatory right heart catheterisation.^14^ Left ventricular (LV) systolic dysfunction was defined as an LV ejection fraction of ⩽45%.

### Cardiac magnetic resonance imaging

#### Protocol

None of the patients had any contraindications for a cardiac MRI, especially renal insufficiency, which has been involved in nephrogenic systemic fibrosis. The examination was performed on a 1.5 Tesla MR scan (Intera, Philips Medical Systems, Best, The Netherlands). After localisation of the four planes of the heart (short axis, SA; long axis, LA; 4 chambers, 4CH; aortic root plane, AR), a turbo spin-echo sequence balanced in T2 black blood in the SA of the heart was performed. A cine-balanced turbo fast echo sequence was performed in three axes (SA, 4CH, AR). After a single injection of 0.1 mmol/kg meglumine gadoterate (Dotarem, Guerbet, Aulnay-sous-Bois, France), perfusion at rest was assessed by an echo-double diffusion imaging sequence in the SA plane. After a second dose of 0.1 mmol/kg meglumine gadoterate, the delayed contrast enhancement sequence was performed in three cardiac axes (SA, LA, 4CH). At the end of the examination a velocimetric sequence centred on the mitral valve was performed.

#### Imaging data analysis

The myocardium was studied in 17 segments according to the American Heart Association standardised myocardial segmentation.^15^ The morphological study assessed the presence of increased intramyocardial signal intensity on T2-weighted images. A thickness of ⩽4 mm was considered as a thinned myocardium. Right ventricular (RV) hypertrophy was defined by a thickness of ⩾5 mm. The presence of LV and/or RV dilatation was defined as an increased indexed LV and/or RV end-diastolic volume compared with available normal values.^16^ ^17^ ^18^ An impaired LV or RV ejection fraction was defined according to normal values provided by Maceira *et al*.^16^ ^18^

Delayed contrast enhancement was defined as an area fulfilling all of the following criteria: a signal intensity value >2 SD above the normal myocardium,^19^ presence in the same myocardial segment in at least two different planes, and presence in identical planes on two different acquisitions with the appropriate inversion time. MRI abnormalities were independently assessed by two expert radiologists (ALH and VG) blinded to the clinical findings. In cases of discrepancy, a consensus was reached by discussion.

### Statistical analysis

All data are presented as mean (SD) or as frequencies (n (%)). Comparisons of means were performed with the non-parametric Wilcoxon test, comparisons of frequencies with the χ^2^ or Fisher exact tests. Correlations between numerical parameters were evaluated using Pearson’s correlation. Statistical analyses were performed with SAS software Version 9.1 (SAS Institute, Cary, North Carolina, USA).

## Results

### Clinical characteristics

The clinical characteristics of the patients are shown in table 1. No patient had overt left heart failure.

**Table 1:** Clinical characteristics of patient population

M/F, n	44/8
Mean (SD) age (years)	56 (11)
Limited/diffuse cutaneous SSc, n	32/20
Anticentromere antibody, n (%)	21 (40)
Anti-SCL 70 antibody, n (%)	18 (34)
Mean (SD) disease duration since first non-Raynaud’s phenomenon (years)	6.6 (6.1)
Mean (SD) disease duration since Raynaud’s phenomenon (years)	11.2 (10.4)
Interstitial lung disease on HRCT, n (%)	25 (48)
Precapillary pulmonary arterial hypertension by right heart catheterisation, n (%)	12 (23)
Palpitations, n	5
Myositis*	0
Hypertension	15
Elevated serum cholesterol levels	15
Current smokers	9
Diabetes mellitus	2
Body mass index >27 kg/m^2^	5
Mean (SD) inspiratory vital capacity (%)	93 (15)
Mean (SD) total lung capacity (%)	95 (14)
Mean (SD) Tlco (%)	59 (25)
Current treatment, n	Calcium channel blockers (n = 15); angiotensin-converting enzyme inhibitors (n = 15); bosentan (n = 12); sildenafil (n = 1)
Prednisone, n (mean (SD) dose in mg)	19 (11 (6))
Previous treatment by cyclophosphamide, n	6

Data are mean (SD) or absolute number (%).

*Based on clinical data and creatine phosphokinase level.

HRCT, high-resolution CT of the chest; SSc, systemic sclerosis; Tlco, carbon monoxide transfer factor expressed as percentage of theoretical value.

### Pattern and distribution of cardiac MRI abnormalities

**Figure 1:**
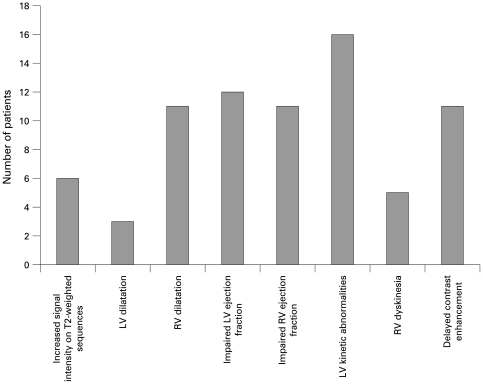
Frequencies of the main MRI abnormalities. LV, left ventricle; RV, right ventricle.

When we excluded mitral flow impairment which was not interpretable in all patients, cardiac MRI showed at least one abnormality in 39 of the 52 patients (75%). The main MRI abnormalities are shown in fig 1.

#### Morphological study

**Figure 2:**
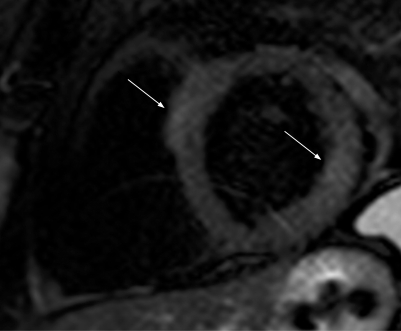
Anteroseptal and lateral (arrows) transmural nodular increased signal intensity in T2-weighted sequence on an MRI 1.5 Tesla scan (Intera, Philips Medical Systems, Best, The Netherlands).

**Figure 3:**
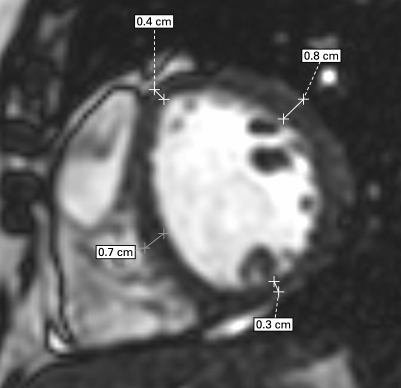
Short axis of the right and left ventricle in cine-MRI sequence showing the thickness of the left ventricle myocardium (end-diastolic frame).

Increased signal intensity on T2-weighted sequences was found in 6 patients (12%, fig 2), always in the LV myocardium and mainly transmural (5 patients). None of these 6 patients had inflammatory markers. The mean (SD) duration of SSc was not significantly lower in patients with increased signal intensity (7.0 (6.3) vs 4.3 (3.8) years, p = 0.28). A thinning of the LV myocardium was observed in 15 patients (29%, fig 3) and predominated in the following segments: 7 (mid-anterior segment) (n = 11), 8 (mid-anteroseptal segment) (n = 9) and 1 (basal anterior segment) (n = 6). No correlation with coronary artery distribution was observed.

LV and RV dilatation were found in 3 patients (6%) with a mean (SD) LV indexed end-diastolic volume of 109 (4) ml/m^2^ and 11 patients (21%) with a mean (SD) RV indexed end-diastolic volume of 99 (32) ml/m^2^, respectively. The RV was hypertrophied in 2 patients (4%). A moderate pericardial effusion was observed in 10 patients (19%).

#### Perfusion analysis

No perfusion defect at rest was detected by visual analysis.

#### Functional study

Twelve of the 52 patients (23%) had an impaired LV ejection fraction (mean (SD) 48 (4)%) and 11 (21%) had an impaired RV ejection fraction (mean (SD) 34 (9)%) without evidence of overt cardiac failure in any patient. LV kinetic abnormalities were found in 16 patients (31%), mainly segmental LV hypokinesia (n = 14) and, more rarely, global LV hypokinesia (n = 2). The abnormalities predominated in segment 7 (mid-anterior segment; n = 9), segment 8 (mid-anteroseptal segment; n = 9) and segment 1 (basal anterior segment; n = 5). Segmental LV dyskinesia was observed in 2 patients and RV dyskinesia was observed in 5 patients (10%).

The transmitral flow was interpretable in 43 of the 52 patients (83%). An impaired LV relaxation pattern was found in 15 of the 43 patients (35%), a normal pattern in 21 patients (49%), a pseudonormal pattern in 6 patients (14%) and a restrictive pattern in 1 patient (2%).

#### Delayed contrast enhancement

**Figure 4:**
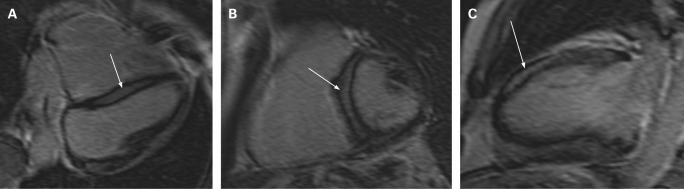
Mid-wall linear late enhancement (arrows) in anteroseptal location assessed by delayed enhancement sequence 10 min after Gd-DTPA injection on an MRI 1.5 Tesla scan. (A) Four chambers; (B) short axis; (C) long axis.

Myocardial delayed contrast enhancement was detected in 11 of the 52 patients (21%). Delayed contrast enhancement was mainly linear (n = 8/11; fig 4) and, more rarely, nodular (n = 3/11). It was mid-wall in the majority of patients (n = 8/11) and, more rarely, subendocardial (n = 2/11) or subepicardial (n = 1/11). It predominated in segments 7 (mid-anterior segment; n = 5), 8 (mid-anteroseptal segment; n = 7) and 9 (mid-inferoseptal segment; n = 6). There was no correlation with any coronary artery distribution.

### Correlation between cardiac MRI abnormalities

Of the 6 patients with increased signal intensity on T2-weighted sequences, one also had myocardial delayed contrast enhancement. Of the 15 patients with thinning of the LV myocardium, 11 had LV kinetic abnormalities. Of the 11 patients with delayed contrast enhancement, 3 had LV kinetic abnormalities. In the first patient the cardiac segment affected by kinetic abnormalities had no concomitant delayed contrast enhancement. In the second patient the segment affected by kinetic abnormalities had delayed contrast enhancement. In the third patient the two segments affected by kinetic abnormalities had delayed contrast enhancement.

### Association between cardiac MRI abnormalities and clinical presentation of SSc

A comparison of cardiac MRI findings between patients with limited cutaneous SSc and patients with diffuse cutaneous SSc is given in table 2, showing no great differences between the two subtypes except for the frequency of impaired LV ejection fraction. A comparison of cardiac MRI findings between patients with and patients without precapillary PAH is shown in table 3. Mean (SD) pulmonary arterial pressure was 34 (13) mm Hg and mean (SD) cardiac index was 2.93 (0.82) l/min/m^2^. All patients with RV dilatation had undergone a right heart catheterisation to exclude the presence of either precapillary PAH or postcapillary pulmonary hypertension. Of the 7 patients with RV dilatation but without PAH, 2 also had LV dilatation. There was a good linear correlation between the cardiac output measured by MRI and by right heart catheterisation (R^2^ = 0.7, p = 0.004).

**Table 2:** Comparison of cardiac MRI findings between patients with limited cutaneous SSc and patients with diffuse cutaneous SSc

	Limited cutaneous SSc(n = 32)	Diffuse cutaneous SSc(n = 20)	p Value
Patients with at least one cardiac MRI abnormality, n (%)	24 (75)	15 (75)	1.00
Morphological abnormalities			
Increased signal intensity in T2-weighted sequence, n (%)	2 (7)	4 (20)	0.20
Thinned LV myocardium, n (%)	8 (25)	7 (35)	0.44
LV dilatation, n (%)	1 (3)	2 (10)	0.55
RV hypertrophy, n (%)	1 (3)	1 (5)	1.00
RV dilatation, n (%)	5 (16)	6 (30)	0.29
Pericardial effusion, n (%)	7 (22)	3 (15)	0.72
Functional abnormalities			
LV kinetic abnormalities, n (%)	10 (31)	6 (30)	0.92
RV kinetic abnormalities, n (%)	3 (9)	2 (10)	1.00
Mean (SD) LV ejection fraction, %	59 (10)	62 (7)	0.20
Impaired LV ejection fraction	11 (34)	1 (5)	0.02
Mean (SD) LV end-diastolic volumes, ml/m^2^	68 (14)	69 (17)	1.00
Mean (SD) cardiac output, l/min	4.8 (1.4)	5.6 (1.3)	0.04
Mean (SD) RV ejection fraction, %	52 (12)	50 (10)	0.31
Impaired RV ejection fraction, n (%)	6 (19)	5 (25)	0.73
Mean (SD) RV end-diastolic volumes, ml/m^2^	73 (11)	87 (28)	0.03
LV diastolic dysfunction on transmitral flow analysis, n (%)	11/29 (38)	4/14 (7)	0.41
Delayed contrast enhancement, n (%)	6 (19)	5 (25)	0.73

Data are mean (SD) or absolute number (%).

LV, left ventricle; RV, right ventricle; SSc, systemic sclerosis.

**Table 3:** Comparison of cardiac MRI findings between patients with and patients without precapillary pulmonary arterial hypertension (PAH)

	Patients with PAH(n = 12)	Patients without PAH(n = 40)	p Value
Patients with at least one cardiac MRI abnormality, n (%)	9 (75)	30 (75)	1.00
Morphological abnormalities			
Increased signal intensity in T2 weighted sequence, n (%)	3 (27)	3 (8)	0.11
Thinned LV myocardium, n (%)	4 (33)	11 (8)	0.72
LV dilatation, n (%)	1 (8)	2 (5)	0.55
RV hypertrophy, n (%)	2 (17)	0 (0)	0.04
RV dilatation, n (%)	4 (33)	7 (17)	0.25
Pericardial effusion, n (%)	5 (42)	5 (13)	0.04
Functional abnormalities			
LV kinetic abnormalities, n (%)	3 (25)	13 (32)	0.73
RV kinetic abnormalities, n (%)	2 (17)	3 (8)	0.32
Mean (SD) LV ejection fraction, %	61 (10)	60 (8)	0.69
Impaired LV ejection fraction, n (%)	3 (25)	9 (23)	1.00
Mean (SD) LV end-diastolic volumes, ml/m^2^	66 (18)	69 (14)	0.39
Mean (SD) cardiac output, l/min	5.6 (1.7)	5.0 (1.2)	0.21
Mean (SD) RV ejection fraction, %	54 (13)	50 (11)	0.20
Impaired RV ejection fraction, n (%)	2 (17)	9 (23)	1.00
Mean (SD) RV end-diastolic volumes, ml/m^2^	75 (9)	79 (23)	0.67
LV diastolic dysfunction on transmitral flow analysis, n (%)	5 (50)	10 (30)	0.49
Delayed contrast enhancement, n (%)	1 (8)	10 (26)	0.42

LV, left ventricle; PAH, precapillary pulmonary arterial hypertension on right heart catheterisation; RV, right ventricle.

Concerning the duration of SSc before cardiac MRI, we found that the longer the disease duration from the first symptom of non-Raynaud’s phenomenon, the greater the number of cardiac segments presenting kinetic abnormalities (r = 0.29, p<0.05) and delayed contrast enhancement (r = 0.30, p<0.05). With Raynaud’s phenomenon as the first sign of SSc, we found that the longer the disease duration, the greater the number of cardiac segments presenting kinetic abnormalities (r = 0.35, p<0.05). No correlation was found with delayed contrast enhancement (r = 0.19, p = 0.15).

### Association between cardiac MRI abnormalities and echocardiography findings

The association between cardiac abnormalities and echocardiographic findings is shown in table 4. The sensitivity of cardiac MRI to detect cardiac abnormalities was 39/52 patients (75%) compared with 25/52 patients (48%) for echocardiography. Of the 3 patients with LV dilatation on MRI, one also had LV dilatation on echocardiography. All of the 11 patients with RV dilatation on MRI had RV dilatation on echocardiography. The mean (SD) LV ejection fraction obtained by echocardiography was significantly higher than that obtained by MRI (63 (7)% vs 60 (9)%, p<0.05). There was a significant but weak linear correlation between LV ejection fraction obtained by these two investigations (r = 0.49, p = 0.001).

**Table 4:** Comparison of cardiac MRI abnormalities and echocardiography findings

	Normal cardiac MRI(n = 13)	Abnormal cardiac MRI(n = 39)
Normal echocardiography (n = 27)	11	16 (7 thinned LV myocardium, 3 LV kinetic abnormality, 3 impaired RV ejection fraction, 2 impaired LV ejection fraction, 2 LV dilatation, 4 delayed contrast enhancement, 2 increased signal intensity in T2-weighted sequence)
Abnormal echocardiography (n = 25)	2 (abnormal thickness of the interventricular septum or posterior wall of the LV)	23

LV, left ventricle; RV, right ventricle.

## Discussion

The main results of our study are as follows. First, the majority (75%) of patients with SSc had at least one abnormality on cardiac MRI which gives a higher sensitivity than echocardiography (48%). Second, cardiac MRI enabled us to analyse precisely the different patterns of heart involvement in SSc by differentiating morphological, functional, perfusion and delayed contrast enhancement abnormalities. Third, patients with limited cutaneous SSc had roughly the same MRI abnormalities as those with diffuse cutaneous SSc, and RV dilatation was not specific for PAH. The high frequency of heart abnormalities observed on cardiac MRI is consistent with necropsy studies which showed that approximately 80% of patients with SSc had histological lesions of heart involvement.^2^ ^3^ As in previous studies,^20^ this complication was rarely detectable at the bedside. Taken together, these results suggest that such alterations are clinically underestimated and that MRI is highly sensitive. Yet the clinical significance of MRI abnormalities remains to be established.

Our study enabled us to distinguish the different patterns of heart involvement in SSc using MRI. Previous studies have shown that MRI can accurately detect myocardial fibrosis.^6^ ^21^ In myocardial fibrosis the gadolinium is trapped in the fibrosis while it is washed more rapidly in the normal myocardium, explaining the delayed contrast enhancement. The myocardial delayed contrast enhancement observed in our study (fig 3) had almost the same characteristics as those in the study by Tzelepis *et al*,^6^ with the same predominance of a mid-wall and linear pattern. Lesions of the small coronary arteries or arterioles were recorded in approximately 20% of necropsy cases.^2^ In chronic infarction, myocardial remodelling results in regional thinning of the myocardium. Thinning of the LV observed in our study could therefore reflect the chronic coronary microvascular injury related to SSc. Finally, inflammation is likely to play a role in SSc as well as in heart involvement.^22^ ^23^ Increased signals on T2-weighted images are indicators of soft tissue oedema.^9^ In the absence of any correlation with coronary artery distribution, increased signal intensity in T2-weighted images is suggestive of inflammatory myocarditis.^24^ ^25^ ^26^

Our study also showed that MRI LV and/or RV ejection fractions were altered in about one-fifth of patients according to the reference values of Maceira *et al*,^16^ ^18^ although the mean values remained within the normal range and patients had no evidence of overt cardiac failure. The alteration of LV and RV ejection fractions is most likely a direct consequence of myocardial fibrosis, as previously suggested.^10^ ^27^

We did not find any perfusion defect on cardiac MRI in patients with SSc. This is consistent with the absence of increased coronary artery arteriosclerosis in SSc.^2^ However, we must acknowledge that our technique may have lacked sensitivity,^11^ precluding the possibility to see perfusion defects usually observed using thallium perfusion scans.^28^

Interestingly, we found no significant differences in cardiac MRI abnormalities between patients with limited cutaneous SSc and those with diffuse cutaneous SSc. These results are consistent with a previous study in which heart symptoms were not found to be significantly different between the two subtypes.^1^ We have recently shown that echocardiographic abnormalities did not differ between diffuse and limited cutaneous SSc.^4^ We found that LV ejection fraction was more often altered in patients with limited cutaneous SSc, although no patients had overt cardiac failure and mean values remained in the normal range.

Up to 17% of the patients in our study without PAH had RV dilatation. It is noteworthy that all these patients underwent right heart catheterisation to rule out PAH. This is further evidence for the specific involvement of the RV in SSc, most probably related to myocardial fibrosis. PAH was rather mild in our study, which probably explains why some patients with PAH had no RV dilatation.

With regard to the comparison of data provided by echocardiography and cardiac MRI, we found that cardiac MRI provides additional information. Some analyses were not possible by echocardiography, most notably delayed contrast enhancement, increased signal intensity and thinned myocardium. However, echocardiography is more useful in valvular heart diseases, especially in PAH screening with tricuspid gradient evaluation. The correlation between LV ejection fraction obtained by MRI and by echocardiography was not good, which has been reported previously.^29^

Our study shows that patients with a longer disease duration had more kinetic abnormalities and delayed contrast enhancement, which is consistent with previous studies.^6^ ^30^ These results suggest a progression of myocardial fibrosis over time and therefore a natural history of heart involvement in SSc. This natural history could be longitudinally studied by repeated cardiac MRI.

We acknowledge that our study has some limitations. There was no histological confirmation of our imaging data since this procedure was judged to be too invasive to be incorporated into the study. We did not include a control group of healthy subjects, thus precluding any firm conclusions regarding the higher frequency of abnormalities. Results from a 3 Tesla MRI scan may have provided more detailed information on the extent of fibrosis and its morphology. We did not systematically measure B-type natriuretic peptide and troponin levels.

Our study shows that MRI is an accurate and reliable technique for diagnosing heart involvement in SSc and for analysing precisely its mechanisms including inflammatory, microvascular and fibrotic components. As it is non-invasive, quantitative and highly sensitive, MRI appears to be the method of choice to determine the natural history of untreated patients or to monitor accurately the effects of treatment. Moreover, it could provide powerful prognostic factors in both groups. Compared with echocardiography, MRI appears to provide additional information by visualising myocardial fibrosis and inflammation. Finally, we have shown that RV dilatation is not specific for PAH and could correspond to a specific heart involvement in SSc. Further studies are required to determine whether cardiac MRI abnormalities have a significant clinical impact on the prognosis and treatment strategy.

## References

[1] Ferri C Valentini G Cozzi F Systemic sclerosis. Demographic, clinical and serologic features and survival in 1012 Italian patients. Medicine 2002;81:139–53 11889413 10.1097/00005792-200203000-00004

[2] D’Angelo WA Fries JF Masi AT Pathologic observations in systemic sclerosis (scleroderma). A study of fifty-eight autopsy cases and fifty-eight matched controls. Am J Med 1969;46:428–40 5780367 10.1016/0002-9343(69)90044-8

[3] Follansbee WP Miller TR Curtiss EI A controlled clinicopathologic study of myocardial fibrosis in systemic sclerosis (scleroderma). J Rheumatol 1990;17:656–62 2359076

[4] de Groote P Gressin V Hachulla E Evaluation of cardiac abnormalities by Doppler echocardiography in a large nationwide multicentric cohort of patients with systemic sclerosis. Ann Rheum Dis 2008;67:31–6 17267515 10.1136/ard.2006.057760

[5] Candell-Riera J Armadans-Gil L Simeon CP Comprehensive noninvasive assessment of cardiac involvement in limited systemic sclerosis. Arthritis Rheum 1996;39:1138–45 8670322 10.1002/art.1780390710

[6] Tzelepis GE Kelekis N Plastiras SC Pattern and distribution of myocardial fibrosis in systemic sclerosis: a delayed enhanced magnetic resonance imaging study. Arthritis Rheum 2007;56:3827–36 17968945 10.1002/art.22971

[7] Vignaux O Allanore Y Meune C Evaluation of the effect of nifedipine upon myocardial perfusion and contractility using cardiac magnetic resonance imaging and tissue Doppler echocardiography in systemic sclerosis. Ann Rheum Dis 2005;64:1268–73 15708883 10.1136/ard.2004.031484 PMC1755644

[8] Friedrich MG Strohm O Schulz-Menger J Contrast media-enhanced magnetic resonance imaging visualizes myocardial changes in the course of viral myocarditis. Circulation 1995;97:1802–9 9603535 10.1161/01.cir.97.18.1802

[9] Garcia-Dorado D Olivears J Gili J Analysis of myocardial oedema by magnetic resonance imaging early after coronary artery occlusion with or without reperfusion. Cardiovasc Res 1993;27:1462–9 8297415 10.1093/cvr/27.8.1462

[10] Bezante GP Rollando D Sessarego M Cardiac magnetic resonance imaging detects subclinical right ventricular impairment in systemic sclerosis. J Rheumatol 2007;34:2431–7 17985401

[11] Allanore Y Meune C Vignaux O Bosentan increases myocardial perfusion and function in systemic sclerosis: a magnetic resonance imaging and tissue-Doppler echography study. J Rheumatol 2006;33:2464–9 17080515

[12] Masi AT Rodnan GP Medsger TA Jr Preliminary criteria for the classification of systemic sclerosis (scleroderma). Subcommittee for scleroderma criteria of the American Rheumatism Association Diagnostic and Therapeutic Criteria Committee. Arthritis Rheum 1980;23:581–90 7378088 10.1002/art.1780230510

[13] LeRoy EC Black C Fleischmajer R Scleroderma (systemic sclerosis): classification, subsets and pathogenesis. J Rheumatol 1988;15:202–5 3361530

[14] Hachulla E Gressin V Guillevin L Early detection of pulmonary arterial hypertension in systemic sclerosis: a French nationwide prospective multicenter study. Arthritis Rheum 2005;52:3792–800 16320330 10.1002/art.21433

[15] Cerqueira MD Weissman NJ Dilsizian V Standardized myocardial segmentation and nomenclature for tomographic imaging of the heart: a statement for healthcare professionals from the Cardiac Imaging Committee of the Council on Clinical Cardiology of the American Heart Association. Circulation 2002;105:539–42 11815441 10.1161/hc0402.102975

[16] Maceira AM Prasad SK Khan M Reference right ventricular systolic and diastolic function normalized to age, gender and body surface area from steady-state free precession cardiovascular magnetic resonance. Eur Heart J 2006;27:2879–88 17088316 10.1093/eurheartj/ehl336

[17] Alfakih K Plein S Thiele H Normal human left and right ventricular dimensions for MRI as assessed by turbo gradient echo and steady-state free precession imaging sequences. J Magn Reson Imaging 2003;17:323–9 12594722 10.1002/jmri.10262

[18] Maceira AM Prasad SK Khan M Normalized left ventricular systolic and diastolic function by steady state free precession cardiovascular magnetic resonance. J Cardiovasc Magn Reson 2006;8:417–26 16755827 10.1080/10976640600572889

[19] Gerber BL Garot J Bluemke DA Accuracy of contrast-enhanced magnetic resonance imaging in predicting improvement of regional myocardial function in patients after acute myocardial infarction. Circulation 2002;106:1083–9 12196333 10.1161/01.cir.0000027818.15792.1e

[20] Maione S Cuomo G Giunta A Echocardiographic alterations in systemic sclerosis: a longitudinal study. Semin Arthritis Rheum 2005;34:721–7 15846587 10.1016/j.semarthrit.2004.11.001

[21] Plastiras SC Kelekis N Tzelepis GE Magnetic resonance imaging for the detection of myocardial fibrosis in scleroderma. N Engl J Med 2006;354:2194–6 16707765 10.1056/NEJMc053128

[22] Roumm AD Whiteside TL Medsger TA Lymphocytes in the skin of patients with progressive systemic sclerosis. Quantification, subtyping, and clinical correlations. Arthritis Rheum 1984;27:645–53 6375682 10.1002/art.1780270607

[23] Liangos O Neure L Kuhl U The possible role of myocardial biopsy in systemic sclerosis. Rheumatology (Oxford) 2000;39:674–9 10888714 10.1093/rheumatology/39.6.674

[24] Codreanu A Djaballah W Angioi M Detection of myocarditis by contrast-enhanced MRI in patients presenting with acute coronary syndrome but no coronary stenosis. J Magn Reson Imaging 2007;25:957–64 17457796 10.1002/jmri.20897

[25] Gutberlet M Spors B Thoma T Suspected chronic myocarditis at cardiac MR: diagnostic accuracy and association with immunohistologically detected inflammation and viral persistence. Radiology 2008;246:401–9 18180335 10.1148/radiol.2461062179

[26] Zagrosek A Wassmuth R Abdel-Aty H Relation between myocardial edema and myocardial mass during the acute and convalescent phase of myocarditis: a CMR study. J Cardiovasc Magn Reson 2008;30:19–27 18447954 10.1186/1532-429X-10-19 PMC2396625

[27] Meune C Allanore Y Devaux JY High prevalence of right ventricular systolic dysfunction in early systemic sclerosis. J Rheumatol 2004;31:1941–5 15468357

[28] Steen VD Follansbee WP Conte CG Thallium perfusion defects predict subsequent cardiac dysfunction in patients with systemic sclerosis. Arthritis Rheum 1996;39:677–81 8630120 10.1002/art.1780390421

[29] Hoffmann R von Bardeleben S ten Cate F Assessment of systolic left ventricular function: a multicentre comparison of cineventriculography, cardiac magnetic resonance imaging, unenhanced and contrast-enhanced echocardiography. Eur Heart J 2005;26:607–16 15618026 10.1093/eurheartj/ehi083

[30] Karwatowski SP Chronos NA Sinclaire H Effect of systemic sclerosis on left ventricular long-axis motion and left ventricular mass assessed by magnetic resonance. J Cardiovasc Magn Reson 2000;2:109–17 11547800 10.3109/10976640009148679

